# Comment on “An Increase in Dietary Supplement Exposures Reported to US Poison Control Centers”

**DOI:** 10.1007/s13181-017-0638-0

**Published:** 2017-11-28

**Authors:** Richard L. Kingston, Andrea W. Wong, Ikhlas Khan

**Affiliations:** 1 SafetyCall International, PLLC, 3600 American Boulevard W, Suite 725, Bloomington, MN 55431 USA; 2 College of Pharmacy, University of Minnesota, Minneapolis, MN USA; 3 Scientific and Regulatory Affairs, Council for Responsible Nutrition, Washington, DC 20036 USA; 4 School of Pharmacy, University of Mississippi, University, MS USA

In the report by Rao et al., the authors’ conclusions raise questions about the safety of dietary supplements (DS); however, there are important study limitations and context that must be considered before drawing conclusions regarding DS safety [1].

The current report purports to review exposures involving the FDA-regulated category of DS products. Yet, several product categories included in the analysis involved products clearly outside the scope of DS, including homeopathic products (representing 36% of the dataset), which are regulated by the US Food and Drug Administration (FDA) as drugs, and beverage versions of energy drinks, which are regulated as foods. Cultural medicines typically include substances that are outside of any regulated category of product. Ma huang products, which accounted for numerous reported exposures, have not been on the market for over a decade and are not relevant to today’s 170 million consumers who take DS as part of their wellness efforts [2].

For those calls that involve exposure to properly categorized DS, it must be emphasized that reports of exposure are not synonymous with adverse events. Parents calling for reassurance subsequent to childhood ingestion of non-toxic amounts of these and other household products are routinely represented in the most recent American Association of Poison Control Centers’ (AAPCC) annual report in which 89% of all pediatric (< 6 years) exposures in the database involve no reports of adverse effects following exposure [3]. For the current report, asymptomatic exposures involve as many as 96% of all pediatric exposures and 99.6% when including minor outcomes.

There are also concerns with the manner in which medical outcomes were further categorized as “serious,” which leads readers to equate those outcomes to life-threatening effects typically requiring life-saving therapeutic interventions, hospitalization, and potential residual disability. Yet, for this report, incidents coded with a moderate outcome were included in the “serious” outcome category. Moderate outcomes within the poison center dataset are, by definition, non-life threatening, leave no residual disability, and can include effects such as mild, self-limiting tachycardia, hives, or rash that require no therapeutic intervention. And, even for those serious events included in the PC data that involve allergy and/or anaphylaxis, confirmation that a DS was the cause with confirmatory allergy testing is rare. Removing the moderate outcome incidents from the report’s serious outcome classification leaves only 0.2% as serious incidents as compared to 4.5% reported by the authors.

Equally concerning is the inclusion of incidents coded as death without some qualification or further review of the relatively small number of such events (*n* = 34). As an example, death associated with ginseng is highly suspect and cannot be independently confirmed, especially when fatality abstracts for this and virtually all reported fatalities presented in the dataset are missing both in this report and the corresponding AAPCC annual reports.

Lastly, trends regarding absolute numbers of calls involving DS exposure are difficult to interpret without a denominator, a shortcoming that has plagued the analysis of poison center calls for decades [4]. Without a corresponding comparison to sales, including trends for symptomatic exposures over time, the analysis lacks critical context.
